# The Poor Man's Home.—VIII

**Published:** 1897-10-09

**Authors:** 


### Modern Sociology.

### THE POOR MAN'S HOME.—VIII.

"Philanthropy and 4 per cent."—(*continued*).

The Glasgow Workmen's Dwellings Company limits its dividends to 5 per cent. As a matter of fact, though the company has earned more, the shareholders have never accepted more than 4 per cent., and the surplus has been spent in various ways to increase the well-being of the tenants. The buildings of the company are called Cathedral Court, and occupy a high and healthy situation near the old cathedral. There are two blocks, one on each side of the court, and the space between is laid out partly with gravel and partly with shrubs. Each block is five stories high and the lodgings are constructed along open galleries. The only objection to this plan is that the family who live at the farther end of the balcony can look into their neighbours' kitchen windows on their way to their own door; but in practice no complaint is made of inconvenience from this cause. Tenants are strictly forbidden to shake mats or carpets over the balconies or to hang out clothes upon them. Laundries and drying space are provided on the roof. There are two tubs and a boiler for each floor, and each tenant has a right to the accommodation belonging to the storey one day a week. Water-closets are situated on the stair landings and one serves for two families. Windows open to the court on one side and to the outside of the building on the other, so that through ventilation is secured. There are 41 two-room tenements, and 17 of one room. The size of the latter is 13 ft. 4 in. by 10 ft., and the rent is 1s. 9d. per week. The two-room tenements have a living-room, the floor space of which is 13 ft. by 10, and a bed-room, which measures 7 ft. by 10. Ceilings are 9 ft. high. The company provides an iron bed for the bed-rooms. The rent is from 2s. 6d. to 2s. 8d. weekly. Twice a year a rebate of a fortnight's rent is allowed to those who have paid their rents promptly in advance. Tenants are under very strict regulations as to the care of the property, and careless or noisy ones can be dismissed. A superintendent lives on the premises, and has power to prevent overcrowding and enforce order. He has also the supervision of the sanitary arrangements, and attends to small repairs.

The society has also bought up some old properties, rearranged them on a more convenient and healthy plan, and let them at a moderate rental—£5 4s. per annum for a one-room, and £7 1s. for a two-room tenement. In each dwelling an iron bed and a kitchen-range is provided.

The Rosemount Buildings in Edinburgh provide for a rather higher rank of artisans. The houses there are built on balconies round a court, like the Glasgow Workmen's Dwellings, but there are no one-room tenements, and the rooms are larger. Two-room tenements have kitchens 14 ft. by 12 in size, and bed-rooms 16 ft. by 12 ft. 10 in. There are three-room tenements of different sizes. In the largest the kitchen is 14 ft. by 12, one bed-room 10 ft. by 10, and the other 10 ft. by 5. In the smaller ones the kitchen is 12 ft. by 12, and the bed-rooms each 7 ft. by 12. Each tenement has a water-closet. The two-room tenements rent at 3s. 3d. per week, and the three-room at from 3s. 6d. to 4s. 7d. per week. As the occupants, as a rule, are artisans earning from £1 to £1 5s. a week these rents might be regarded as consuming an undue proportion of the income; but, although rents must be paid quarterly in advance, a failure to pay rent is almost unknown. The company pays 6 per cent. dividend.

Well Court Tenements, Edinburgh, are meant for a higher class of tenants, although the accommodation is much the same as that in the Rosemount Buildings—two or three rooms. Rents range from £7 to £11 9s., but the property pays only a little over 3 per cent. The building, however, is artistic, and each tenement, besides being complete and admirable from a sanitary point of view, is painted, papered, and supplied with grates and gas-fittings.

The examples given here may be taken as showing what private enterprise can do in providing for the housing of the working classes when carried on under unselfish, yet practical, management. In only one case, that of Manchester, are operations carried on at a loss; it is to be regretted that there is any, but we do not know enough of the special circumstances to say whether or not this unsatisfactory condition of things can be remedied. But we think that the other examples abundantly prove that it is possible for private companies to build houses for the poor which shall meet their needs and yet give a reasonable return on capital. This, we consider, is the ideal to be aimed at. To give workers dwellings at an unremunerative rate is, according to all the laws of political economy, to make it possible for them to work for lower wages. The benefit is not in the end theirs, but their employers. Their greater advantages tend in the result to lower the status of their fellows.

For this reason we have said nothing of the work of the Peabody and Guinness Trusts in London. Yet, as carried on, these cannot be regarded as, in the degrading sense of the word, charitable. The Peabody buildings earn about 3¼ to 3½ per cent. on the capital expended; but this, not being distributed, is added to the capital fund for the further development of the operations of the Trust. As for this purpose the Peabody trustees have borrowed money, the profit derived from rents is devoted to paying interest on this, and repaying capital. The rent per room is 2s. 1¾d. This is higher than in several other towns, but the high ground rents of London must be considered. The average earnings of the head of each family are £1 3s. 6d. per week; thus 20 per cent. of his earnings go in rent. This seems excessive, but as similar accommodation in the neighbourhood costs 25 per cent. more, the Peabody Buildings are eagerly sought after.

In the Guinness Trust Buildings an even higher proportion of the income is paid in rent, but the officials declare that the poorest tenants are the most reliable rent-payers. The Guinness tenements really cater for the poorest. One-room tenements rent at from 1s. 6d. to 2s. 6d. per week, and larger dwellings are from 3s. to 5s.; but an extra charge (3d. a week for one room, and 6d. a week for two and three-room tenements) is made for the use of baths and club-room, for hot-water supply, and chimney-sweeping. This might be thrown into the rent, but the notion of paying for it as an extra charge—it is compulsory—induces many tenants to make use of the baths and club-room who would not otherwise do so.

